# Book Notices

**Published:** 1869-09

**Authors:** 


### Book Notices.

Circular No. 2, War Department of Surgeon General's Office. Report on Excisions of the Head of the Femur for Gun Shot Injury. Washington: Government Printing Office. 1869.

This is a report of so much of the Surgical History of the late war as relates to Excisions of the Head of the Femur, for Gun Shot Injuries. It is prefaced with a historical review of this operation, and throughout beautifully illustrated. In the qualities of paper, type, and illustration, it certainly does credit to the Department. We have not had time to examine its contents, but have no doubt of their great value, especially to every practical Surgeon.

A Treatise on Diseases of the Eye. By J. Soelberg Wells, Professor of Ophthalmology in King's College, London; Ophthalmic Surgeon to King's College Hospital, and Assistant Surgeon to the Royal London Ophthalmic Hospital,

Moorfields. First American Edition, with Additions. Illustrated with 216 engravings on wood, and six colored plates, together with selections from the Test-Types of Prof. E. Jaeger and Dr. H. Sneller. Philadelphia: Henry C. Lea. 1869.

This is a full-sized octavo of 736 pages, substantially bound in leather, and on fair type and paper. It is intended to constitute a complete treatise on diseases, injuries, and defects of the organs of vision. This will be found one of the best among many works relating to the same department of medicine and surgery.

---

Report of the Board of Health of New York for the year 1867.

This is a full-sized octavo volume, of 635 pages, with many valuable illustrations. To those interested in sanitary science and hygiene it is a volume of special interest. To the ordinary practitioner it furnishes many facts calculated to increase his knowledge of the etiology of diseases, and consequently of the means for their prevention. It contains a very full and interesting account of the disease called the "Cattle Plague," in all its relations.

We presume copies can be obtained by addressing the Secretary of the Board, Emmons Clark, New York.

